# Preventive Supplementation of Omega-3 Reduces Pain and Pro-inflammatory Cytokines in a Mouse Model of Complex Regional Pain Syndrome Type I

**DOI:** 10.3389/fnint.2022.840249

**Published:** 2022-03-30

**Authors:** Taynah de Oliveira Galassi, Paula Franson Fernandes, Afonso Shiguemi Inoue Salgado, Francisco José Cidral-Filho, Anna Paula Piovezan, Daniela Dero Lüdtke, Josiel Mileno Mack, Kenneth A. Weber, William R. Reed, Franciane Bobinski, Daniel F. Martins

**Affiliations:** ^1^Experimental Neuroscience Laboratory (LaNEx), Postgraduate Program in Health Sciences, University of Southern Santa Catarina - UNISUL, Palhoça, Brazil; ^2^Natural Quanta Wellness Center, Windermere, FL, United States; ^3^Department of Medical Clinic, Graduate Program in Medical Sciences, Federal University of Santa Catarina - UFSC, Florianopolis, Brazil; ^4^Division of Pain Medicine, Stanford School of Medicine, Palo Alto, CA, United States; ^5^Department of Physical Therapy, University of Alabama at Birmingham, Birmingham, AL, United States

**Keywords:** pain, CRPS, ischemic reperfusion, omega-3 polyunsaturated fatty acids, complex regional pain syndrome (CRPS)

## Abstract

Complex regional pain syndrome type I (CRPS-I) is a condition that responds poorly to treatments. The role of omega-3 fatty acids in the treatment of inflammatory disorders is well described in the literature; however, few studies have evaluated its therapeutic benefits in different types of pain. We evaluated the potential antihyperalgesic and anti-inflammatory effects of preventive omega-3 supplementation in an animal model of CRPS-I. In experiment 1, Swiss female mice were supplemented for 30 days with omega-3 before the induction of the CRPS-I model and 14 days after. Mechanical hyperalgesia was evaluated at baseline and from the 4th to the 14th day after CPRS-I induction along with open field locomotor activity after 30 days of supplementation. In experiment 2, Swiss female mice were supplemented for 30 days with omega-3 and then subjected to the CRPS-I model. Twenty-four hours later the animals were euthanized, and tissue samples of the spinal cord and right posterior paw muscle were taken to measure pro-inflammatory cytokine TNF and IL-1β concentrations. Omega-3 supplementation produced antihyperalgesic and anti-inflammatory effects, as well as reducing pro-inflammatory cytokine concentrations, without altering the animals’ locomotion. No open field locomotor changes were found. The 30-day supplementation at the tested dose was effective in the CRPS-I model.

## Introduction

Complex regional pain syndrome-Type I (CRPS-I) is defined as a disabling chronic clinical condition, that often develops after minor or severe trauma, and responds very poorly to a variety of current treatments (Midbari et al., 2016). Patients typically present with sensory, autonomic, and motor abnormalities, as well as acute inflammatory features such as pain, redness, warmth, and edema in the affected limbs. CRPS-I can develop after any kind of injury, including crush injuries and/or muscle sprains. It is estimated that approximately 1% of all fractures will develop into CRPS-I, although it is most prevalent in cases of distal radius fractures (4–37% of cases) with lesser triggers including carpal tunnel surgery (2–4% of cases) and surgery to correct Dupuytren’s contracture (Al Sayegh et al., 2013). CRPS-I is marked by the severity of the symptoms, which are disproportionate to the severity of the trauma, with a tendency to spread in the affected distal limb (Wasner et al., 1998). While pathophysiological mechanisms of CRPS-I remain controversial, evidence indicates that inflammatory and sympathetic nervous system mechanisms are involved (Coderre and Bennett, 2010).

In CRPS, it is hypothesized that trauma to the limb triggers an exacerbated inflammatory process that exceeds the clinical course and expected duration of the initial injury (Coderre and Bennett, 2010). CRPS-I symptoms include spontaneous pain and pain induced by what would otherwise be considered innocuous stimuli (mechanical and cold allodynia) as well as increased sensitivity to painful stimuli (mechanical or thermal hyperalgesia) (Lee et al., 2018). Patients present with vasomotor and sudomotor dysfunction, as well as trophic changes in skin, hair, nails, and bones (Coderre and Bennett, 2010; Lee et al., 2018). Current treatment involves pharmacological interventions (tricyclic antidepressants, anticonvulsants, opioids, anti-inflammatory drugs, and corticosteroids), physical therapy, psychotherapy, chemical and surgical sympathectomy, and interventional blockade of the sympathetic nervous system (Lee et al., 2018). Despite the wide variety of clinical interventions currently employed, CRPS-I treatment remains challenging with poor clinical efficacy overall (Dworkin et al., 2003).

The role of omega-3 polyunsaturated fatty acids (PUFA), especially eicosapentaenoic acid (EPA) and docosahexaenoic acid (DHA), is well described for the prevention and treatment of inflammatory disorders (Nobre et al., 2013). Proposed mechanisms of action include the deviation of eicosanoid synthesis to non-inflammatory products, the production of anti-inflammatory lipid mediators (such as resolvins, protectins, and maresins), and inhibition of nuclear factor kappa B (NF-κB) activation. Despite the proven anti-inflammatory effects of omega-3 fatty acids, their therapeutic benefits in different types of pain have not yet been demonstrated (Zhang et al., 2017). In this context, considering the anti-inflammatory properties of omega-3 PUFAs, as well as the various benefits provided to individuals when supplemented, we hypothesized that omega-3 supplementation could serve as an alternative or possible adjunct treatment to CRPS-I to eliminate or minimize common side effects often associated with conventional drug treatment.

Given the inflammatory response, pain, and similar CRPS features induced after prolonged ischemia and reperfusion in the mouse model of CRPS-I [9], and the anti-inflammatory activity of omega-3 PUFAs, the purpose of this study was to use behavioral and biochemical tests to evaluate the antihyperalgesic and anti-inflammatory effectiveness of omega-3 supplementation in a mouse model of CRPS-I.

## Materials and Methods

### Animals

The present study was conducted at the Experimental Neuroscience Laboratory (LaNEx)–University of the South of Santa Catarina (UNISUL). Swiss female mice (approximately 2 months of age), weighing between 30 and 40 g were obtained from the central animal care facility of the Federal University of Santa Catarina (UFSC). The animals were kept at 22 ± 2°C, in a 12 h light/dark cycle, with access to food and water *ad libitum*. All animals received the same standard NUVILAB CR-1 diet. The animals were acclimated in the laboratory 1 h before the experiments. Animals with baseline response frequency above 20% in the evaluation of mechanical hyperalgesia were excluded from the study. Mice were allocated in the housing boxes and distributed across the experimental groups of 9–12 animals, in a randomized and blinded manner. The research was approved by UNISUL Ethics Committee on Animals Use (CEUA) under protocol No. 18.050.4.01.IV. All experiments were performed according to the Laboratory Animal Care Guide and Ethical Guidelines for Experimental Investigations of pain in conscious animals (Zimmermann, 1983).

### Chronic Post-ischemic Pain

The chronic post-ischemic pain CRPS-I animal model was induced by prolonged exposure of the animal’s right paw to ischemia, followed by reperfusion, as previously described (Millecamps et al., 2010). The mice were anesthetized with an intraperitoneal injection of thiopental (80 mg/kg, intraperitoneal [i.p]) and an elastic band (Elastic band 000–1237, Uniden) with 1.2 mm internal diameter was placed close to the ankle joint of the right posterior paw, thereby decreasing blood flow. After 3 h the band was removed, allowing paw reperfusion. Anesthetic supplementation was administered to animals that began to regain consciousness prior to the stipulated 3-h period (thiopental, 20% of the initial volume at the same dose concentration).

### Omega-3 Supplementation

Fish oil supplementation was performed by intragastric (i.g.) route using Natural Quanta omega-3 (Orlando, FL, United States). Each 1,000 mg capsule contained 400 mg of EPA and 300 mg of DHA. The animal received a daily treatment of 1,500 mg/kg, i.g. (EPA: 600 mg; DHA: 450 mg), as previously described (Lobo et al., 2016). For this study, two experiments were carried out, using the following groups: (1) Sham–No Ischemia and Reperfusion (IR) + Saline (10 mL/kg, i.g.); (2) Control group–IR + Saline (10 mL/kg, i.g.); (3) IR + Omega-3 (1.5 g/mouse, i.g.); and (4) Control group–IR + Corn Oil (1.5 g/mouse, i.g.).

**FIGURE 1:**
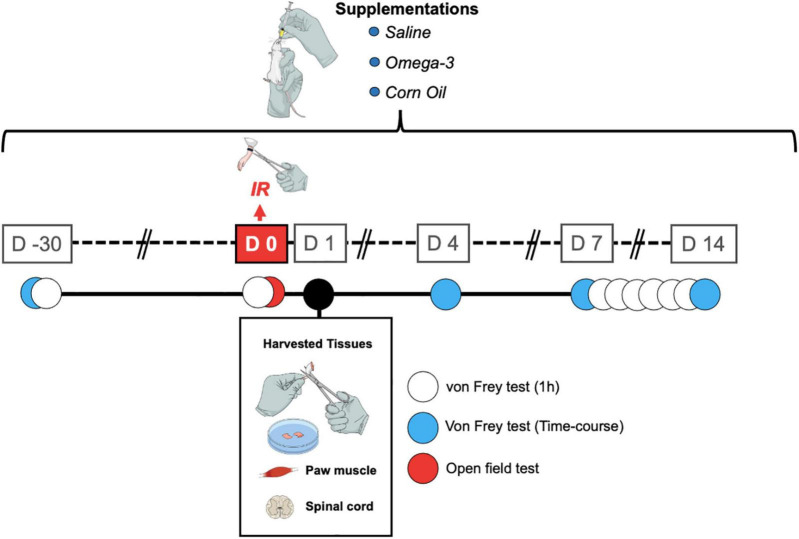
Timeline showing the treatments and assessments. IR, ischemia and reperfusion; D, day.

The first experiment consisted of conducting behavioral tests with a supplementation protocol of 45 days, and the second experiment was aimed at analyzing the concentrations of pro-inflammatory cytokines with a supplementation protocol of 30 days (Figure 1).

### Experiment 1: Behavioral Tests

#### Mechanical Hyperalgesia

Mechanical hyperalgesia was assessed by blinded experimenter using von Frey monofilaments (VFH, Stoelting, Chicago, IL, United States), with a 0.6 g load, as previously described (Martins et al., 2015). The animals were acclimated individually in the laboratory 1 h before the experiments in a bottomless acrylic box (9 cm × 7 cm × 11 cm) and then placed on a wire mesh platform allowing access to the plantar surface of the animals’ paw. Paw withdrawal frequency to 10 right hind paw non-consecutive stimulations with a von Frey monofilament (0.6 g) were recorded as an index of mechanical hyperalgesia.

The day before the start of omega-3 supplementation, the animals were evaluated to obtain the baseline response. The time course analyses of the antihyperalgesic effects induced by omega-3 supplementation were performed on days 4, 7, and 14 following the IR procedure and 1, 2, and 3 h after omega-3 supplementation. In a separate set of experiments, mechanical hyperalgesia was assessed every day following omega-3 supplementation between the 7th and 14th day after the IR procedure, always 1 h after omega-3 supplementation (Figure 1).

#### Open Field Test

In order to evaluate the effects of omega-3 supplementation alone and the possible induction of a sedative effect, spontaneous locomotion was assessed using the open field test. The apparatus consists of a square wooden arena 40 cm × 40 cm × 40 cm in size. The animals were placed individually in the center of the apparatus and were allowed to freely explore it for a period of 5 min (Eysenck, 1960). The animal’s exploratory behavior was filmed. The total walking distance traveled (in meters), and the maximum speed (meters/seconds) were assessed by blinded experimenters using ANYmaze^®^ software (Stoelting, United States). The apparatus was cleaned with a 10% ethanol solution between tests to avoid signs of animal odor. The evaluations were carried out at the times described in Figure 1.

### Experiment 2: Biochemical Analyzes

#### Enzyme-Linked Immunosorbent Assay

For collection of biological samples, all animals were euthanized 24 h after IR and 2 h after omega-3 supplementation (Figure 1). Right paw muscle plantar flexor and spinal cord (L3–L6) tissue samples were removed with the aid of a forceps and a surgical blade. After dissection, the samples were immediately frozen in liquid nitrogen and stored in a freezer at −80°C until tissue analyses. The tissues were homogenized with phosphate-buffered saline (PBS) with Tween-20 (0.05%), phenylmethylsulfonyl fluoride (PMSF, 0.1 mM), ethylenediaminetetraacetic acid (EDTA, 10 mM), aprotinin (2 ng/ml), and benzethonium chloride (0.1 mM). The homogenate was transferred to Eppendorf tubes, centrifuged at 3,000 × *g* for 10 min at 4°C. The supernatant was stored at −80°C for future analysis (Bobinski et al., 2011). The amount of protein in the supernatant was measured by the Bradford method (Bradford, 1976) using bovine serum albumin as standard. A volume of 100 μL of the sample was used to measure TNF and IL-1β levels using enzyme-linked immunosorbent assay (ELISA) kits for mice (Invitrogen/Thermo Fisher Scientific, Waltham, MA, United States) according to the manufacturer’s instructions. Cytokines levels were estimated by interpolation from a standard curve by colorimetric measurements at 450 nm (correction wavelength 540 nm) in an ELISA plate reader (Perlong DNM-9602, Nanjing Perlove Medical Equipment Co, Nanjing, China). The processing and analysis of the samples were performed by an evaluator blinded to the groups and experimental interventions. All results were expressed as pg/mg of protein.

### Statistical Analysis

Results are presented as the mean ± standard deviation (SD) for each group. Initially, the Shapiro-Wilk normality test was applied to evaluate the normality of the data. Mechanical hyperalgesia was analyzed using two-way repeated measures ANOVA. A multiple comparison *post-hoc* test was performed using Bonferroni correction. Open field test and cytokines levels were analyzed using between-subjects *t*-tests. Differences with a value of *P* < 0.05 were considered statistically significant. Statistics were calculated using Graph-Pad Prism 5.0 software (San Diego, CA, United States).

## Results

### Effects of Omega-3 Supplementation on Mechanical Hyperalgesia

**FIGURE 2:**
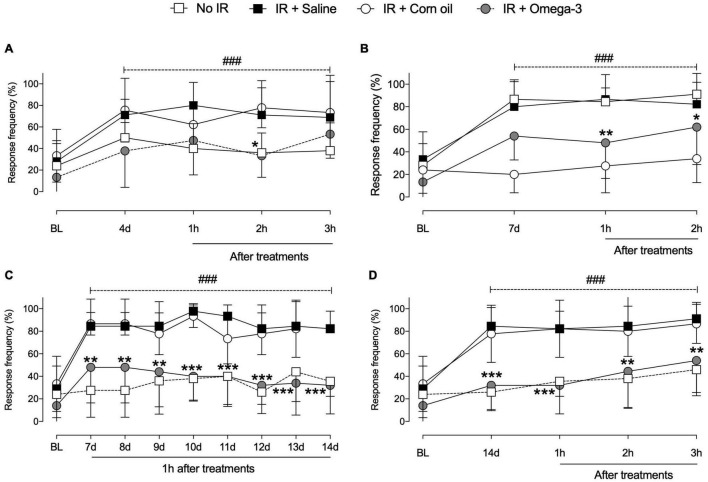
Effects of preventive Omega-3 supplementation on mechanical hyperalgesia. Time course evaluations on day 4 [panel **(A)**], day 7 [panel **(B)**], and day 14 [panel **(D)**]. Evaluations 1 h after daily supplementation between the 7th and 14th day after IR procedure [panel **(C)**]. Each dot represents mean of 8–10 animals and the vertical lines show the standard deviation (SD). Statistical analyses performed by two-way ANOVA followed by Bonferroni test. The symbols indicate a significant difference of *P < 0.05, **P < 0.01, and ***P < 0.0001 when comparing the IR + saline vs. IR + Omega-3 groups or ^###^*P* < 0.0001 when comparing the No IR vs. IR + saline groups. IR, ischemia-reperfusion.

As shown in Figure 2, the IR procedure induced hyperalgesia, verified by increased paw withdrawal response frequency (*P* < 0.001) when compared to No IR (sham) animals. On the 4th day, significant differences were observed between groups (IR + Saline vs. IR + Omega-3), 2 h (*P* = 0.008) after omega-3 supplementation (Figure 2A). On the 7th day post IR, an antihyperalgesic effect was observed up to 2 h after supplementation (*P* = 0.03, Figure 2B).

According to daily evaluations (from the 7th to the 14th day following IR), omega-3 supplementation decreased mechanical hyperalgesia when compared to IR + Saline (*P* < 0.001, Figure 2C). Temporal course evaluations indicated that preventive omega-3 supplementation produced a reduction in mechanical hyperalgesia from the 14th day after the IR procedure onward, even before daily treatment, indicating sustained antihyperalgesic effects between omega-3 doses (3 h, *P* < 0.0001, Figure 2D). Corn oil supplementation induced no antihyperalgesic effects. These animals showed no changes in mechanical hyperalgesia after the IR procedure when compared to saline-supplemented animals (Figures 2A–D).

### Effects of Omega-3 Supplementation on Open Field Locomotor Activity

**FIGURE 3:**
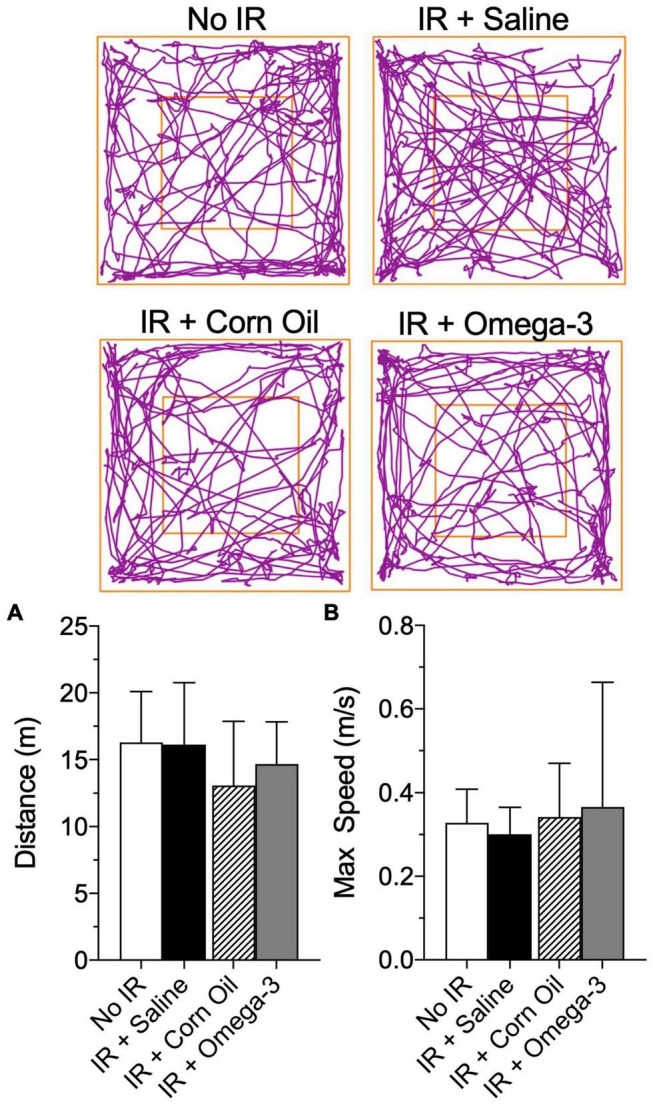
Effect of preventive Omega-3 supplementation on locomotor activity. Representative figures of the route in the open field test (superior panels), distance traveled [meters, panel **(A)**] and maximum speed reached [meters/seconds, panel **(B)**]. Vertical bars represent mean of 10 animals and vertical lines show standard deviation (SD). Statistical analyses performed by one-way ANOVA followed by Bonferroni test.

No changes were observed in traveled distance (meters) or maximum speed (meters/seconds) when comparing omega-3 with the saline group prior to the IR procedure (Figure 3). In addition, there were no changes in distance traveled or maximum speed when comparing the animals supplemented with corn oil to those from the saline group (Figure 3).

### Effects of Omega-3 Supplementation on Pro-inflammatory Cytokines

**FIGURE 4:**
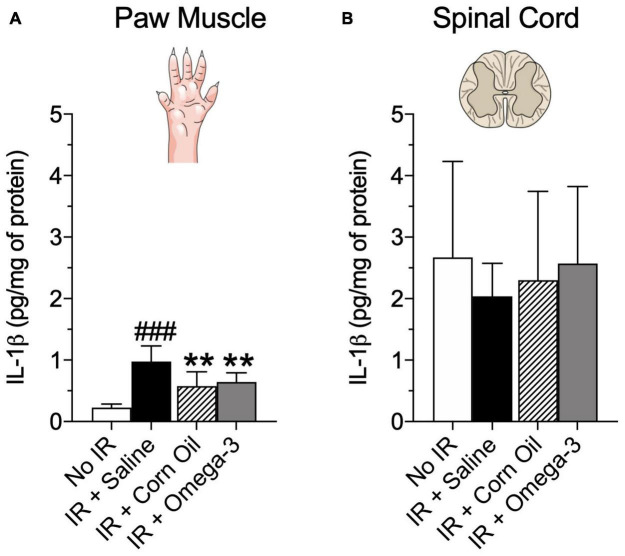
Effects of preventive Omega-3 supplementation on pro-inflammatory cytokine IL-1β concentrations. IL-β concentrations in paw muscle [panel **(A)**] and spinal cord [panel **(B)**]. Each dot represents mean of eight animals and the vertical lines show the standard deviation (SD). Statistical analyses performed by one-way ANOVA followed by Bonferroni test. The symbols indicate a significant difference of **P < 0.01 when comparing the IR + Saline vs. IR + Omega-3 groups or ^###^*P* < 0.001 when comparing the No IR vs. IR + Saline groups. IR, ischemia-reperfusion.

As shown in Figure 4, **24** h after the IR procedure, the IR group presented increased IL-1β concentration in paw muscle when compared to the No IR group (*P* = 0.001, panel A). Conversely, the omega-3 supplementation (*P* = 0.03) and corn oil group (*P* = 0.01), displayed lower IL-1β levels after the IR procedure when compared to the IR + Saline group (panel A). No changes were found in spinal cord IL-1β levels (*P* = 0.26, Figure 4B).

**FIGURE 5:**
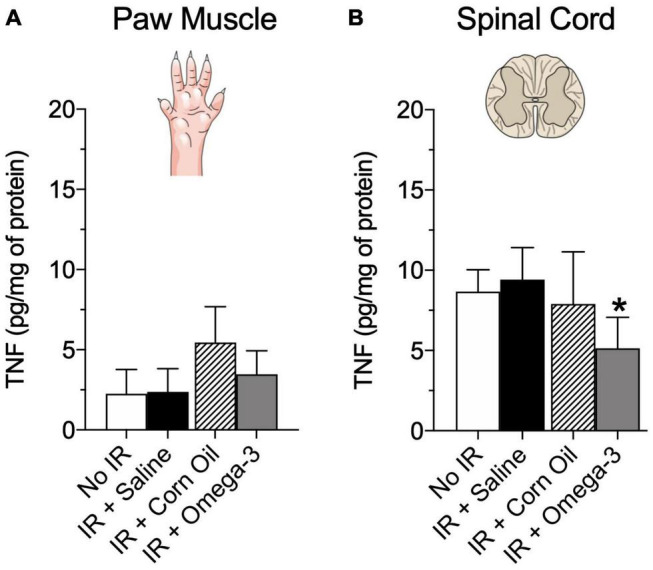
Effects of preventive Omega-3 supplementation on pro-inflammatory cytokine TNF concentrations. TNF concentrations in paw muscle [panel **(A)**] and spinal cord [panel **(B)**]. Each dot represents mean of 6–7 animals and the vertical lines show the standard deviation (SD). Statistical analyses performed by one-way ANOVA followed by Bonferroni test. The symbols indicate a significant difference of *P < 0.05 when comparing the IR + Saline vs. IR + Omega-3 groups. IR, ischemia-reperfusion.

Moreover, Figure 5 demonstrated that the IR + omega-3 group also exhibited lower concentrations of spinal cord TNF when compared to the IR + Saline group (*P* = 0.03, panel B). No changes were found in paw muscle TNF concentrations (*P* = 0.89, Figure 5A).

## Discussion

In this study, we corroborate and extend earlier findings that 30-day preventive omega-3 supplementation protocol induces prolonged cumulative antihyperalgesic effects, resulting in reduction in mechanical hyperalgesia and produced anti-inflammatory effects by reducing the production of pro-inflammatory cytokines in both peripheral and central sites in an animal model of CRPS-I. The benefits of omega-3 PUFAs in preventing and treating inflammatory disorders are well established (Schwanke et al., 2016; Zhang et al., 2017; Pauls et al., 2018). However, there is lack of evidence for possible anti-inflammatory properties of omega-3 PUFAs in animal models of CRPS-I or in human CRPS clinical trials. In addition, the effect of omega-3 supplementation on neuropathic pain in general has been poorly studied (Redivo et al., 2019). Hence, the present study was the first in the literature to use a 30-day preventive omega-3 supplementation protocol, which mimics clinical use, in a mouse CRPS-I model. Specifically, we evaluated the antihyperalgesic potential of preventive omega-3 supplementation in an animal model of CRPS-I in both the acute/inflammatory as well as the chronic/neuropathic phase of the disease (Coderre and Bennett, 2010; Klafke et al., 2016).

Here, we observed that the IR procedure, an accepted model of CRPS-I [9], induced mechanical hyperalgesia in the acute (hot/inflammatory) and chronic (cold/neuropathic) CRPS phases. The key finding of this study was that preventive omega-3 supplementation reduced CRPS-related hyperalgesia from the 4th day after IR onward, showing a cumulative and lasting effect elicited by supplementation through the 14th day.

The observed effects of omega-3 supplementation on the inflammatory and neuropathic phases of CRPS-I clinical features are consistent with some previous studies in other pain conditions. For example, mice supplemented for 21 days with a fish oil-enriched diet prior to pain induced by hemorrhagic cystitis, showed a reduction in mechanical allodynia when compared to corn oil supplemented animals (Freitas et al., 2015), corroborating current results in the acute inflammatory phase of CRPS-I. In addition, the fact that omega-3 preventive supplementation reduced mechanical hyperalgesia in the CRPS-I neuropathic phase in our study corroborates (Redivo et al., 2019) prior results in a diabetic neuropathy model, which found that acute omega-3 supplementation administered 15 or 30 days after neuropathy development decreased mechanical allodynia. Furthermore, 7 days of omega-3 supplementation produced lasting and cumulative antihyperalgesic effects in this latter animal model (Redivo et al., 2019). Also in the diabetic neuropathy model, mice treated for 60 days with a fish oil-enriched diet displayed reduced mechanical hyperalgesia (Obrosov et al., 2017), and that preventive omega-3 supplementation for 60 days effectively decreased hyperalgesia at all doses tested (Redivo et al., 2016).

Even though there are several studies showing omega-3 activity in reducing inflammatory nociceptive pain and some neuropathic pain states as noted above (Lu et al., 2013; Nobre et al., 2013; Freitas et al., 2015), it should be noted that investigations regarding omega-3 effects in the well-established partial sciatic nerve ligation model of neuropathic pain are somewhat contradictory. Using this model, omega-3 supplementation for 5 days significantly reduced mechanical allodynia, producing antihyperalgesic effects at 3 h following supplementation which was maintained for up to 24 h (Silva et al., 2017). On the other hand, other work using this neuropathic pain model found that supplementation for 14 days with oils containing different concentrations of omega-3 or -6 showed no effect on mechanical allodynia (Pérez et al., 2005). In summary, our results confirm that preventive omega-3 supplementation reduces mechanical hyperalgesia specifically in CRPS-I and adds to the literature by demonstrating these beneficial effects in both the inflammatory and neuropathic phases of an animal model of CRPS-I.

The omega-3 supplementation dose used here was defined according to previous non-clinical studies (in rodents) and inflammatory and neuropathic pain models (Nobre et al., 2013; Redivo et al., 2019). The protocol of preventive supplementation, by intragastric route, of 30 days was based on the literature and considering common clinical use of omega-3 PUFAs (Redivo et al., 2016). We note that chronic omega-3 supplementation, at the dose used, did not induce any adverse effects, i.e., diarrhea, regurgitation, changes in weight or behavior, when compared to animals in the control group (data not shown). We chose corn oil for supplementation in the control group based on previous studies which also used it due to its inert state and lack of omega-3 actions (Pérez et al., 2005; Freitas et al., 2015). s Chronic painful conditions, including CRPS, are usually sustained by peripheral and/or central sensitization (Latremoliere and Woolf, 2009; Bruehl, 2015; Ji et al., 2018). These phenomena are produced in part by the action of pro-inflammatory cytokines that alter the activity of neuronal ion channels, leading to hyperresponsiveness (Hu et al., 2010; Klafke et al., 2016). Pro-inflammatory cytokines such as IL-1β and TNF play an important role in the genesis of neuronal sensitization and, consequently, in the pathophysiology of chronic painful conditions. In the present study, we found an increase in IL-1β concentrations in the paw muscle following IR during the acute phase (day 1). These findings agree with previous studies that have demonstrated the importance of peripheral inflammatory processes (peripheral sensitization) in the mechanical hyperalgesia observed in CRPS-I, showing the increase of IL-1β concentration in the peripheral tissue of rats after IR only in the acute phase (2 and 48 h) (Laferrière et al., 2008; de Mos et al., 2009). Although it has been suggested that TNF also contributes to the pathogenesis of CRPS-I after peripheral IR (Sabsovich et al., 2008; Klafke et al., 2016), we did not find changes in the concentration of this cytokine in paw muscles in the acute phase (day 1). These findings may be related to the fact that there is a window where there is an increase in some cytokines and not others in sites such as the paw and spinal cord. Thus, future studies with different analysis times and cytokines can further clarify these questions. However, we observed that preventive omega-3 supplementation decreased IL-1β, but not TNF concentrations, in paw muscles. This is perhaps not surprising given that we only observed post-IR increases in IL-1β. Since we could not find similar studies analyzing the effects of preventive omega-3 supplementation on IL-1β and TNF concentrations of paw muscle, it was not possible to make direct comparisons with existing literature.

It is well documented that activation of microglia and astrocytes in the spinal cord after peripheral nerve or tissue injury plays an important role in inducing and maintaining chronic pain (Ji et al., 2018), mechanisms that likely to contribute to CRPS as well. Thus, inhibition of microglial activation may prevent the development of neuropathic pain. In addition, the action of TNF and IL-1β on their respective receptors expressed on microglia and astrocyte membranes may potentiate the activation of these cells, perpetuating chronic pain. To address this possible CRPS mechanism, we measured the concentrations of IL-1β and TNF in the spinal cord. We observed that animals receiving preventive omega-3 supplementation had lower spinal TNF concentrations after the IR procedure when compared to the control group. Corroborating our findings, it has also been shown that animals submitted to partial sciatic nerve ligation and 10-day oral supplementation of concentrated fish oil had lower concentrations of TNF in the spinal cord when compared to vehicle-treated mice 9 days after neuropathy induction (Silva et al., 2017). On the other hand, intrathecal DHA pre-injection was found to decrease both IL-1β and TNF (*via* decreased microglial activation) in the spinal cord of mice with inflammatory pain, highlighting the effects of omega-3 on activated microglia (Lu et al., 2013).

As previously described, NF-κB transcription is also involved in the pathophysiology of CRPS-I. CRPS-I patients who exhibit IR signals that can induce NF-κB activation mediated by ROS and peroxynitrite formation (de Mos et al., 2009), which consequently may induce the production of inflammatory cytokines such as IL-1β and TNF. Interestingly, it has been shown that one of the mechanisms of action related to the anti-inflammatory effects of omega-3 supplementation is NF-κB inhibition (Ji et al., 2011), which may explain the observed effects on pro-inflammatory cytokine concentrations in the current work. The results obtained by Liu et al., 2016 support our findings in the spinal cord, i.e., intrathecal administration of resolving D1 (pro-resolving lipid mediator derived from omega-3) in a neuropathic pain model led to a decrease in pro-inflammatory cytokines IL-1β and TNF, concomitantly with a decrease in NF-κB/p65 activation in the spinal cord and dorsal root ganglia.

Finally, the present study demonstrated that chronic 30-day omega-3 supplementation elicited antihyperalgesic and anti-inflammatory properties without interfering with open field locomotor activity, since the animals supplemented with omega-3 did not show significant differences when compared to control groups. Our findings support other studies showing that both acute (Silva et al., 2017) or chronic (Redivo et al., 2016) omega-3 supplementation does not affect locomotion, highlighting the pain and inflammation-specific effects of omega-3 PUFAs.

Taken together, we conclude that preventive omega-3 supplementation is effective in reducing mechanical hyperalgesia in both the inflammatory and neuropathic phases of CRPS-I. In addition, omega-3 supplementation produced anti-inflammatory effects by reducing the production of pro-inflammatory cytokines in both peripheral and central sites without altering the animals’ locomotion. Thus, this preclinical study provides evidence for the rational use of preventive omega-3 supplementation in future randomized controlled trials. For example, it may be worthwhile to investigate whether omega-3 supplementation reduces risk of CRPS after common injuries known frequently to trigger CRPS, such as distal radius fracture. In the present study, only female mice were used to reduce sex-related variability in pain response and because CRPS is more frequent in females in the proportion of 3:1. The choice to exclude males may be considered a limitation of the study and may reduce generalizability of the findings to male mice (Bussa et al., 2015; Pieretti et al., 2016).

In summary, in a preclinical model of CRPS-I in mice, preventive omega-3 supplementation induced prolonged cumulative antihyperalgesic effect resulting in a reduction in mechanical hyperalgesia. The findings of the present study support previous findings in the literature in other pain conditions and demonstrate potential benefits of omega-3 supplementation for the acute (inflammatory) and chronic (neuropathic) phases of the CRPS-I model. It also indicates a effective dose of omega-3 supplementation in mice for the condition studied. The benefits of omega-3 supplementation may extend beyond CRPS-I and may prove to be an effective therapy for other painful inflammatory and neuropathic conditions.

## Data Availability Statement

The original contributions presented in the study are included in the article/supplementary material, further inquiries can be directed to the corresponding author.

## Ethics Statement

This study was conducted according to the Laboratory Animal Care Guide and Ethical Guidelines for Experimental Investigations of pain in conscious animals. UNISUL Ethics Committee on Animals Use (CEUA) under protocol No. 18.050.4.01.IV.

## Author Contributions

TO: conceptualization, methodology, validation, formal analysis, investigation, data curation, and writing – original draft. PF: methodology, formal analysis, writing – original draft. AS and FC-F: methodology, resources, and review and editing. AP: methodology. DL, KW, and WR: review and editing. JM: methodology, formal analysis, and investigation. FB: term, conceptualization, methodology, validation, formal analysis, resources, review and editing, and supervision. DM: term, conceptualization, methodology, validation, formal analysis, resources, writing – original draft, supervision, funding acquisition. All authors contributed to the article and approved the submitted version.

## Author Disclaimer

The content is solely the responsibility of the authors and does not necessarily represent the official views of the National Institutes of Health.

## Conflict of Interest

The authors declare that the research was conducted in the absence of any commercial or financial relationships that could be construed as a potential conflict of interest.

## Publisher’s Note

All claims expressed in this article are solely those of the authors and do not necessarily represent those of their affiliated organizations, or those of the publisher, the editors and the reviewers. Any product that may be evaluated in this article, or claim that may be made by its manufacturer, is not guaranteed or endorsed by the publisher.

## Abbreviations

CRPS: complex regional pain syndrome
CRPS-I: complex regional pain syndrome type I
PUFA: polyunsaturated fatty acids
EPA: eicosapentaenoic acid
DHA: docosahexaenoic acid
NF-κB: nuclear factor kappa B
LaNEx: experimental neuroscience laboratory
UNISUL: University of the South of Santa Catarina
UFSC: Federal University of Santa Catarina
CEUA: Ethics Committee on Animals Use
IR: ischemia and reperfusion
i.p: intraperitoneal
i.g.: intragastric
PBS: phosphate-buffered saline
PMSF: phenylmethylsulfonyl fluoride
EDTA: ethylenediaminetetraacetic acid
ELISA: enzyme-linked immunosorbent assay
SD: mean ± standard deviation.
